# The Role of Protein Arginine Methylation in mRNP Dynamics

**DOI:** 10.4061/2011/163827

**Published:** 2011-04-07

**Authors:** Michael C. Yu

**Affiliations:** Department of Biological Sciences, State University of New York at Buffalo, 109 Cooke Hall, Buffalo, NY 14260, USA

## Abstract

In eukaryotes, messenger RNA biogenesis depends on the ordered and precise assembly of a nuclear messenger ribonucleoprotein particle (mRNP) during transcription. This process requires a well-orchestrated and dynamic sequence of molecular recognition events by specific RNA-binding proteins. Arginine methylation is a posttranslational modification found in a plethora of RNA-binding proteins responsible for mRNP biogenesis. These RNA-binding proteins include both heterogeneous nuclear ribonucleoproteins (hnRNPs) and serine/arginine-rich (SR) proteins. In this paper, I discuss the mechanisms of action by which arginine methylation modulates various facets of mRNP biogenesis, and how the collective consequences of this modification impart the specificity required to generate a mature, translational- and export-competent mRNP.

## 1. Introduction

In eukaryotic cells, transcription is central to control of the level of gene expression, which, in turn, is critical for normal cell growth, development, and cellular differentiation. During eukaryotic transcription, the nascent pre-mRNA associates with a myriad of RNA-binding proteins, allowing a series of RNA processing steps to take place in a transcription-dependent manner (reviewed in [1, 2]). These processing steps include 5′-end capping, splicing, 3′-end cleavage, and polyadenylation. Many factors involved in these steps physically and functionally associate with the carboxy-terminal domain (CTD) of RNA polymerase II (pol II) (reviewed in [3, 4]). A number of extensively studied RNA-binding proteins function in pre-mRNA splicing and the intracellular transport of mature and stable mRNAs. These include members of both the serine/arginine-rich (SR) proteins and heterogeneous nuclear ribonucleoproteins (hnRNP) families (reviewed in [5, 6]). Once transcription is complete, the mature message is coated with a unique complement of RNA-binding proteins, forming a messenger ribonucleoprotein particle (mRNP) that is then exported out of the nucleus for translation of the RNA component in the cytoplasm. mRNPs in the process of assembly are checked for export competence by nuclear surveillance mechanisms, and any aberrant mRNP is subject to control by such a mechanism. One example of such a mechanism is the nonsense-mediated decay (NMD) pathway, which retains defective mRNPs to be degraded, thereby preventing them from being translated (reviewed in [7, 8]). Given the coupling between particular steps of transcription and specific RNA processing events, mRNP biogenesis must be carried out in a dynamic and coordinated manner, and this is accomplished by a series of molecular recognition events during transcription. 

Protein arginine methylation is a type of post-translational modification that has been identified in many of the factors that are involved in the production of a mature, translatable mRNP, and has emerged as a major regulator of protein function in mRNP dynamics. This particular modification is catalyzed by members of an enzyme family known as protein arginine methyltransferases (PRMTs) (reviewed in [9, 10]). To date, ten mammalian PRMTs have been identified based on their primary sequence and substrate specificity, with six displaying type I activity (PRMT1, PRMT2, PRMT3, PRMT4, PRMT6, and PRMT8) and two displaying type II activity (PRMT5, PRMT7). PRMT7 has also been shown to display type III activity. All of the identified PRMTs use S-adenosyl-*L*-methionine (AdoMet) as the methyl donor in the methyltransferase reaction. The type I PRMTs transfer either one or two methyl groups from AdoMet to a single guanidino nitrogen on a protein-incorporated arginine residue, thus forming either a monomethylarginine (MMA) or an asymmetric dimethylarginine (aDMA). Type II PRMTs also catalyze monomethylation; however, they then proceed to add the second methyl group to the opposing guanidino nitrogen within the arginine residue, thereby forming a symmetric dimethylarginines (sDMA). Among eukaryotes, PRMT1 is the most highly conserved type I PRMT, and PRMT5 is the most highly conserved type II PRMT. Homologs of mammalian PRMTs can be found in trypanosomes, one of the earliest diverged eukaryotes. The preferred methylation target sequence for PRMT1 is an arginine residue flanked by one or more glycine residues (e.g., RGG) [11, 12], although findings from *in vitro* substrate profiling suggest that this enzyme may have additional target sequences [13]. Many of the RNA processing factors involved in mRNP biogenesis contain “RGG” boxes, domains known to participate in both protein-protein and protein-RNA interactions (reviewed in [14]). The aDMA-generating enzymes PRMT3, PRMT6, and PRMT8 also mostly methylate GAR (glycine and arginine-rich) motifs; to date, PRMT4 (also known as CARM1) has no known preferred motif. The sDMA-generating enzyme PRMT5 methylates arginines that are either next to or within GAR motifs. These general rules regarding methylation sites are subject to many exceptions, and the identification of additional PRMT substrates may warrant a revision of these notions in the future. The compendium of RNA processing factors that contain mono- and dimethylated arginines, as established by analysis using both arginine dimethyl-specific antibodies and proteomic identification technologies, is substantial [15, 16]. However, in many cases, it remains to be determined how these methylated arginines influence the molecular activities of many of the affected RNA-binding proteins. In this review, I will discuss our current understanding of how arginine methylation impacts mRNP biogenesis.

## 2. Arginine Methylation in the Maturation of U-snRNPs

An important facet of mRNP biogenesis is the splicing of pre-mRNAs (reviewed in [17]). The macromolecular machinery that carries out pre-mRNA splicing is called the spliceosome, a complex comprised of five U small nuclear ribonucleoprotein particles (snRNPs) and a large number of non-snRNP proteins (reviewed in [18, 19]). In mammalian cells, each spliceosomal snRNP is first assembled in the cytoplasm from a seven-membered ring of core Sm proteins (SmB/B', SmD1, SmD2, SmD3, SmE, SmF, and SmG) and a newly exported snRNA (U1, U2, U4, or U5). The mature snRNPs in the cytoplasm are then imported into the nucleus, where they function in pre-mRNA splicing (reviewed in [20]). A properly assembled Sm core is required for the nuclear import of mature cytoplasmic U-snRNPs. Once in the nucleus, the U-snRNPs are further processed in Cajal bodies before becoming incorporated into a functional spliceosome, and it is the U-snRNP that enables the spliceosome to carry out the catalytic step in pre-mRNA splicing (reviewed in [21]).

The initial clue that suggested a role for arginine methylation in U-snRNP function was the finding of sDMAs on the C-terminal RG dipeptide repeats of the Sm proteins SmD1 and SmD3 [22]. The survival of motor neurons (SMN) protein, which is required for the cytoplasmic assembly of U-snRNPs (reviewed in [23, 24]), preferentially binds to dimethylarginine-modified RG domains of SmD1 and SmD3 [25], demonstrating that this modification is functionally significant in U-snRNP biogenesis. The Luhrmann group further showed that SmB/B' and Sm-like protein LSm4 also contain sDMAs, and that symmetric dimethylation of the four Sm/Sm-Like proteins (SmD1, SmD3, SmB/B', and LSm4) facilitates their interaction with the SMN protein [26]. The interaction between the Sm proteins and SMN requires SMN's Tudor domain, a structural motif that consists of approximately 60 amino acids [27] and is now recognized as a methyl-amino-acid-binding protein module [28, 29]. Using an sDMA-specific antibody, the Richard group immunoprecipitated coilin and the Sm proteins Sm B/B' and SmD [30]. In addition, they showed that arginine methylation is required for the localization of SMN to Cajal bodies [30].

PRMT5 has been linked to the methylation of Sm proteins by studies showing that a protein complex called the methylosome, which incorporates PRMT5 along with Sm proteins and pICln, catalyzes sDMA formation on the Sm proteins [31, 32]. PRMT5 may accomplish this by binding to the RG domains of SmD1 and SmD3 [31]. pICln is known to bind to the Sm domain of Sm proteins, and negatively regulates snRNP assembly [32, 33]. Reconstitution experiments *in vitro* showed that PRMT5 promotes, in an ATP-dependent manner, the direct transfer of Sm proteins by the SMN complex onto the U-snRNAs [34], consistent with the finding that small interfering RNA-(siRNA-) mediated PRMT5 knockdown correlates with decreased recognition of SmB/B' and SmD epitopes by the sDMA-specific antibody [30]. 

 Together, these data demonstrate that PRMT5 is the enzyme that methylates the Sm proteins, and that methylation provides the signal needed for Sm proteins to target the SMN complex for the assembly of snRNP core particle. Given that proper, functional pre-mRNA splicing requires mature U-snRNPs, it would not be surprising to find that defects leading to incorrectly assembled U-snRNP compromise the splicing of pre-mRNAs. Indeed, depletion of sDMA-containing proteins from mammalian nuclear extracts by an sDMA-specific antibody impairs pre-mRNA splicing and formation of the spliceosomal complex [30]. In *Arabidopsis*, loss of the PRMT5 homolog AtPRMT5 leads to widespread defects in pre-mRNA splicing [35]. In *Drosophila*, the PRMT5-homolog Dart5 plays a role in regulating alternative splicing of specific target mRNAs [36]. 

Recent evidence has pointed to the involvement of additional PRMTs in the biogenesis of Sm proteins. A study by the Matera group showed that human PRMT7 is capable of catalyzing sDMA formation in Sm proteins, independent of PRMT5 activity [37]. This mechanism may be functionally conserved in *Drosophila*, as both its PRMT5 and PRMT7 homologs (Dart5 and Dart7, resp.) are required for catalyzing sDMA formation on the Sm proteins [38]. However, whereas Dart5-mediated methylation of Sm proteins is required for an efficient interaction between Sm and SMN, the modification is not required for snRNP biogenesis, as it is in mammalian cells [37]. Rather, Dart5-mediated methylation of Sm proteins is essential for germ-cell specification and maintenance [39]. It is possible that the underlying cause of this difference in the requirement for PRMT5-mediated methylation of Sm proteins in the assembly of snRNPs is differences in the mechanism of snRNP assembly in the two systems. Further complicating our understanding of the role of arginine methylation is the fact that whereas aDMA is present on SmD1, SmD3, and SmB/B' proteins purified from mammalian nuclear fractions, it is sDMA that is present on the same proteins purified from the cytoplasm [40]. While the functional significance of aDMA on these Sm proteins remains to be determined, this observation suggests that an additional aDMA-catalyzing PRMT, possibly PRMT4/CARM1 [41, 42], is present in the snRNP maturation pathway.

## 3. Arginine Methylation of hnRNPs

As RNA Pol II transcription commences, many hnRNPs are recruited to the nascent transcript for the purpose of regulating the life cycle of the mRNA (reviewed in [5]). The hnRNP proteins vary vastly in terms of domain composition and functional properties, with some promoting mRNP biogenesis by effecting pre-mRNA splicing and others the packaging of mRNPs for nuclear export. The general characteristics of some hnRNPs include RNA-binding domains, RGG boxes with interspersed aromatic amino acids and auxiliary domains such as GAR domains. While the bulk of hnRNP proteins localize predominately to the nucleus at steady state, they undergo some nucleocytoplasmic shuttling, movement that is thought to aid in the transport of mRNAs out of the nucleus, as well as to serve certain cytoplasmic functions.

Early studies of partially purified hnRNP core complexes in mammalian cells revealed the presence of dimethylarginines in hnRNP A and B of this complex [43, 44]. Using both *in vitro* and *in vivo* methylation assays, the Dreyfuss group showed that many hnRNPs are arginine methylated, including hnRNPs E, G, H, J, K, P, Q, R, and U [45]. Many of these hnRNPs were subsequently confirmed to contain mono- and dimethylated arginines, using a proteomic method called SILAC (Staple Isotope Labeling by Amino acids in Cell culture) [16]. This study by the Mann group also identified the same modification in additional RNA-binding proteins, and in hnRNPs that were previously not known to be methylated [16]. PRMT1 is the PRMT that catalyzes this modification in many hnRNPs [46–48], but others have been implicated [49]. The association of PRMT1 with hnRNP particles may be mediated through one of its substrates, hnRNP U [50], a multifunctional nucleic-acid-binding protein that is also known as scaffold attachment factor A (SAF-A) and to have roles in transcription and RNA maturation [51, 52]. However, it is not clear how arginine methylation affects the function of hnRNP U.

One of the functional consequences of arginine methylation for many hnRNPs is their relocalization within the cell. In mammalian cells, arginine methylation facilitates nuclear import or slowing of nuclear export of these hnRNPs. This role is supported by the observation that the suppression, in the cases of hnRNP A2 and Q, of arginine methylation leads to a shift from predominately nuclear localization to predominately cytoplasmic localization [46, 47, 53].

Treatment with the methyltransferase inhibitor adenosine dialdehyde (AdOx) resulted in increased cytoplasmic localization of Src substrate-associated during mitosis (Sam68), an RNA-binding protein that belongs to the hnRNP K homology (KH) domain family [54]. In contrast with observation from mammalian systems, arginine methylation of the budding yeast *S. cerevisiae* hnRNP-like proteins Npl3, Hrp1, and Nab2 promotes their proper nuclear export [55, 56]. Npl3 is a major yeast mRNA export factor and possesses characteristics of both hnRNP and SR-family proteins [57–59]. In yeast cells carrying a specific mutation of Npl3, the presence of the budding yeast homolog of mammalian PRMT1, Hmt1, is required for cell viability [60]. Hrp1 is the subunit of cleavage factor I that is responsible for the cleavage and polyadenylation of pre-mRNA 3′-ends [61]. Nab2 is necessary for efficient nuclear export of bulk mRNAs [62]. A detailed mutagenesis study on methylated arginines within Npl3 showed that methylation is important for its nuclear export, and that the methylation state of Npl3 is linked to the export of Hrp1 [63, 64]. Substitution mutations that change methylated arginines within Hrp1 to lysines are not sufficient to block its nuclear export, further supporting the notion that methylated Npl3 is required for the export of Hrp1, possibly as a part of the mature mRNP complex [64]. Npl3 is also phosphorylated, and this modification controls its nuclear import and is countered by arginine methylation [57, 65]. This example underscores the possibility that a protein's function can be regulated combinatorially by multiple post-translational modifications. In sum, the consequences of arginine methylation are generally changes in the subcellular localization of hnRNPs, but with exceptions, such as for the arginine methylation of mammalian hnRNP A1 and L and of the budding yeast protein Hrb1 [56].

A number of studies have shown that arginine methylation of an hnRNP compromises its ability to interact with nucleic acids. For example, arginine methylation of hnRNP A1 reduced its ability to bind to nonspecific single-stranded nucleic acids [66]. Arginine methylation of Sam68 and its homologues SLM-1 and SLM-2 (Sam68-like mammalian proteins) impairs their ability to bind to poly(U) RNA [67], and arginine methylation of Aly/REF leads to a decrease in its association with mRNA *in vivo* [68]. However, there are examples where arginine methylation does not affect the association of hnRNP with RNA [69–71]. For example, Hrp1 is arginine methylated only when it is not bound to RNA, as prior Hrp1 binding to RNA with a single UAUAUA element blocks Hmt1-mediated methylation [71]. Thus, how arginine methylation modulates the binding capacity of an hnRNP for nucleic acids appears to be influenced by substrate rather than being a general phenomenon.

## 4. Arginine Methylation of SR Proteins

The SR proteins, which are highly conserved at both the functional and structural levels, are non-snRNP splicing factors that have been proposed to play a role in the early stages of spliceosome assembly (such as during the recruitment of U1 snRNP and U2AF to the 5′ and 3′ ss), and to facilitate formation of the U4/U6.U5 tri-snRNP complex during the later steps of splicing (reviewed in [6]). In addition, the SR proteins have been demonstrated to affect each step of post-transcriptional regulation (reviewed in [72]). The SR proteins have a modular domain structure, consisting of one or two RNA-recognition domains (RRM) and a C-terminal rich in arginine/serine residues (RS-rich domain). The RRM domain binds RNA, whereas the RS-rich domain modulates both interactions with other proteins and interactions with pre-mRNA sequences. Currently, two SR proteins have been identified that contain methylated arginines: SFRS9/SRp30c [73] and SF2/ASF [74].

The human SFRS9/SRp30c has been implicated in splicing control, based on the observation that interaction of SFRS9/SRp30c with Tra2*α*, another SR-like protein, promotes splicing of the gonadotropin-releasing hormone pre-mRNA [75]. SFRS9/SRp30c attenuates the repressive effect of a downstream U1 snRNP binding site by stimulating the splicing event to a downstream 5′-ss [76]. In human HEK-293 cells, EGFP-tagged SFRS9/SRp30c accumulates in the nucleoli upon treatment with AdOx, and has suggested that SFRS9/SRp30c may play a role in maturation of snRNAs, as these small RNAs transit through the nucleoli before reaching the nuclear speckles [73]. 

SF2/ASF is a shuttling, broad-specificity splicing regulator with additional roles in NMD [77], mRNA export [78], and the regulation of translation [79]. Previous studies have demonstrated that the phosphorylation of SF2/ASF controls its subcellular localization [80]. In a recent report, the Krainer group showed that methylation of three arginines located in the linker region of SF2/ASF regulates its subcellular localization, as an SF2/ASF mutant carrying substitution mutations that convert all three arginines into alanines displayed increased cytoplasmic accumulation [74]. Whether arginine methylation of SF2/ASF affects its phosphorylation, or vice versa, has not been determined. In these specific triple-alanine mutants, *in vitro* and *in vivo* splicing were compromised, and enhancement of NMD was no longer observed [74]. The splicing defects observed are attributed to the change in the subcellular levels of SF2/ASF, as restoring the levels of the triple-alanine mutant in the nucleus to the wild-type level rescued the splicing defects, consistent with the previous observation that SF2/ASF regulates splicing in a concentration-dependent manner [74, 81]. However, the SF2/ASF triple-alanine mutant was not able to enhance NMD despite the increase in its concentration in the nucleus [74].

## 5. Arginine Methylation of Pre-mRNA Splicing Factors

It is well established that the coupling of transcription with mRNA processing is critical for mRNP biogenesis (reviewed in [82]). Notably, whereas some of the factors are exclusive for one process or the other, others such as mammalian CA150 participate in both [83]. CA150 is methylated by both PRMT4/CARM1 and PRMT5, and its methylation by PRMT4/CARM1 promotes its interaction with the Tudor domain of SMN [42, 84]. It is thought that methylation of CA150 by PRMT4/CARM1 promotes exon skipping in a methyltransferase enzyme-dependent manner [42]. Additionally, PRMT4/CARM1 methylates three splicing factors, SmB, U1-C, and SAP49 [42]. In a separate study, the association of U1-C with an isoform of PRMT4/CARM1 was found to affect 5′-ss selection during pre-mRNA splicing, albeit in a nonenzymatic-dependent manner [85]. A recent study showed that PRMT4/CARM1 is automethylated, and regulation of alternative splicing is impaired in PRMT4/CARM1 automethylation-defective mutants [86]. Thus, it appears that PRMT4/CARM1 is capable of regulating pre-mRNA splicing through more than one mechanism. 

*In vivo *studies of spliceosome recruitment to actively transcribed genes have delineated the order of assembly, with the U1 snRNP being the first component recruited to a newly formed 5′ ss on the transcript, followed by the U2 and U5 snRNPs (reviewed in [87]). In mammalian cells, the U1 snRNP is composed of U1-A, U1-C, and U1-70K proteins (reviewed in [88]). Proteomic and immunological analyses have demonstrated that U1-70K contains dimethylated arginines [15, 89]. However, the PRMT responsible for this modification remains to be determined. In *S. cerevisiae*, the U1-70K homolog Snp1 interacts with Hmt1 by two-hybrid analysis and is an *in vitro* substrate of Hmt1 [89, 90]. ChIP-chip studies have shown that the loss of either Hmt1 or its catalytic activity affects the cotranscriptional recruitment of a number of pre-mRNA splicing factors to intron-containing genes [89]. The mechanistic explanation for this observation is that loss of Hmt1's catalytic activity strengthens the protein-protein interaction between Snp1 and Npl3 [89]. In mammalian cells, the exon junction complex (EJC) is positioned on spliced mRNA in a sequence-independent manner during the pre-mRNA splicing reaction [91]. Deposition of the EJC is also implicated in a number of postsplicing events such as mRNA export, NMD, and the control of translation (reviewed in [7, 92]). The EJC component Y14 can be methylated by PRMT1 *in vitro* [93]. Like Npl3, Y14 is phosphorylated, and this phosphorylation is antagonistic to its methylation [93]. A recent study by the Tarn group indicated that Y14 interacts with the cytoplasmic PRMT5-containing methylosome to promote its methylation of the Sm protein [94].

## 6. Mechanisms by which Arginine Methylation Modulates mRNP Dynamics

Since the assembly of mRNPs during transcription is a rapid and ordered process, each of the factors recruited has the potential to influence downstream events depending on where and when the factor acts. Therefore, post-translational modification of mRNP components may provide the molecular switch needed to allow mRNP components to act dynamically, in concert with each other and in a timely manner. Given the uniqueness of each mRNP complex, large-scale genomic methods have provided the holistic view of how arginine methylation impacts the *in vivo* recruitment of mRNP components to their targets during mRNP biogenesis. In yeast cells lacking Hmt1, the pattern of co-transcriptional recruitment of Npl3 and the transcriptional elongation factor Tho2 is similar to that in wild-type cells [95]. However, the recruitment of downstream mRNP components such as Hrp1, Nab2, and Yra1 differs to various degrees [95]. Biochemical analysis indicates that methylation is likely to provide the “off” switch needed for Npl3 and Tho2 to interact with one another, and that failed disengagement of these two factors is likely the reason for the observed change in the subsequent mRNP dynamics [95]. In these cells, binding between Npl3 and Snp1 is also affected [89]. However, in this case, the loss of methylation results in their failure to dissociate, which likely explains the compromised co-transcriptional recruitment of U2 and U4/5/6-tri snRNPs in the Hmt1 mutants [89]. In addition to controlling protein-protein interactions between mRNP components, arginine methylation modulates the ability of certain RNA-binding proteins to target their nucleic acid substrates, as this modification increases hydrophobicity without changing the positive charge of the arginine [96]. In addition, a substantial loss of hydrogen bonding from methylation can also alter the binding affinity of the protein [97]. For many of the hnRNPs, methylated arginines have been found in regions with a known RNA-binding motif. Indeed, inhibition of methylation at the arginine-glycine rich region of the fragile X mental retardation protein (FMRP) reduces its ability to bind its target mRNA [98]. Such reduced binding, however, is sometimes the normal outcome. For example, methylation of the mammalian mRNA export factor REF/ALY reduces its RNA-binding capacity to ensure that the message can be efficiently displaced by a second mRNA export factor known as TAP [68]. 

Given the importance of coupling between transcription and RNA processing events in the context of mRNP biogenesis, it would not be surprising to find that arginine methylation of components exerts an effect (possibly via a feedback loop) on either the transcription or RNA-processing machinery to preserve proper mRNP dynamics. As previously mentioned, arginine methylation of CA150 influences the coupling of transcription and pre-mRNA splicing [42]. In addition, methylation of hnRNP K enhances the transcriptional activity of tumor suppressor p53 [99]. With regard to the reverse direction, studies in *S. cerevisiae* have shown that changes in transcriptional elongation play a role in Hmt1-dependent recruitment of mRNA export factor Npl3 [100]. Recent studies have also connected chromatin modification to the regulation of alternative splicing [101] and, since multiple PRMTs modify histones, histone modification is one potential area in which arginine methylation could impact RNA processing events. 

It can be inferred that a change in the cellular localization of proteins such as hnRNPs and SR proteins would lead to defects in RNA trafficking since the majority of these proteins are bound to mRNAs. Indeed, in cells treated with a methyltransferase inhibitor, Sam68 fails to export unspliced HIV viral RNAs [54]. The hnRNP A2 is implicated in intracellular transport of the myelin basic protein (MBP) mRNA. In cells treated with AdOx, cytoplasmic granules containing hnRNP A2 are changed and this change correlates with the presence of MBP mRNA granules close to the nucleus [53]. A similar mode of regulation has been implicated in regulation of the Ewing sarcoma RNA-binding protein EWS [102], the *Xenopus* cold-inducible RNA-binding protein CIRP2 [103], and RNA helicase A [104, 105]. However, in the case of RNA helicase A, arginine methylation modulates nuclear import as opposed to the nuclear export as in the case of most hnRNPs and SR proteins [106]. 

As transcription terminates, arginine methylation of the remaining bound processing proteins modulates subsequent steps, promoting further maturation and stability for translation in the cytoplasm. For example, in mammalian cells a subunit of the mammalian pre-mRNA cleavage factor I (CF I_m_68) can be methylated by both PRMT1 and PRMT5 *in vitro* [107]. However, the functional significance of this modification on CF I_m_68 function remains to be investigated. Arginine methylation has also been identified in poly(A)-binding protein family members, including PABP1 [12] and PABP2 [108]. Poly(A)-binding proteins are involved in the synthesis of poly(A) tails (reviewed in [109]). PABPN1, the mammalian nuclear poly(A)-binding protein, can be methylated by PRMT1, −3, and −6 [110]. Unlike PABPN1, the PABPN1 homolog in the fission yeast* Schizosaccharomyces pombe* (termed Pab2) is methylated only by the PRMT1 homolog, Rmt1 [111]. Loss of arginine methylation results in increased Pab2 self-association or aggregation [111]. This phenotype, however, is not observed with the mammalian PABPN1 [110] The mammalian Hu proteins belong to a family of highly conserved RNA-binding proteins that help stabilize mRNAs prior to translation (reviewed in [112]). HuR and HuD, two members of this family, are *in vitro* substrates of PRMT4/CARM1 [113, 114]. Arginine methylation of these two proteins regulates their binding to mRNA, but in the opposite manner: methylation of HuR further stabilizes the binding to the SIRT1 mRNA, whereas methylation of HuD reduces its association with the p21 mRNA [115, 116].

## 7. Concluding Remarks and Future Perspectives

**Figure 1:**
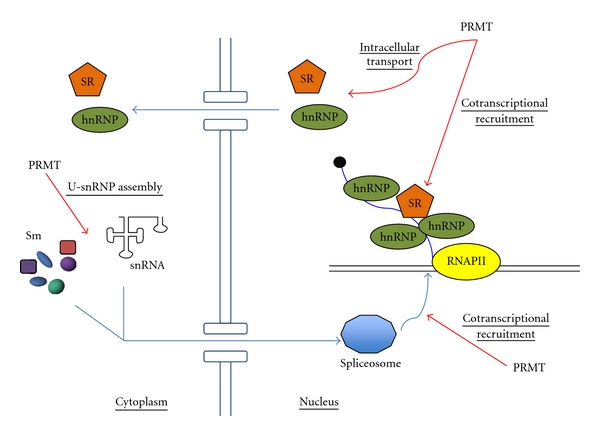
The effects of protein arginine methylation on mRNP dynamics. Arginine methylation affects the assembly of U-snRNPs, components that constitute the spliceosome. The subcellular localization of both SR and hnRNP proteins are modulated by arginine methylation. Loss of arginine methylation also results in co-transcriptional recruitment changes during mRNP biogenesis.

Understanding how arginine methylation modulates mRNP dynamics has provided important insights into how post-translational modifications impact mRNP biogenesis. Nevertheless, the available data on the functional consequences of this common modification for mRNP dynamics remain largely descriptive. From our current understanding, a number of themes are beginning to emerge (Figure 1): (1) arginine methylation leads to a change in the subcellular concentration of an mRNP component, thereby controlling its function. This type of regulatory mechanism is well demonstrated for SF2/ASF, although its relevance to hnRNP-mediated promotion of mRNP biogenesis remains unclear. (2) Arginine methylation leads to a change in the capacity of the protein to recognize either other proteins or nucleic acids. A perturbation of this type would lead to a perturbation in the intricate number of functional connections with various RNA processing factors, as well as the transcriptional machinery, thereby allowing a different set of proteins to be recruited to a nascent transcript and altering the fate of the mRNP. This mechanism enables arginine methylation to signal transition from one stage of mRNP biogenesis to the next at multiple interfaces, for example, between transcriptional and RNA processing machineries (as in the case of CA150); among distinct RNA processing machineries (as in the case of the interaction between Npl3 and Snp1); among components within large, macromolecular machineries important for mRNP biogenesis (as for U-snRNP assembly). (3) Arginine methylation promotes combinatorially, in conjunction with other post-translational modifications. An example is the phosphorylation and arginine methylation of Npl3, which control either nuclear import or export. Additionally, combinatorial regulation with other post-translational modifications such as ubiquitination may serve as a way to reverse the effects of methylation. At this point, knowledge of other post-translational modifications on mRNP components is needed to assess this possibility. 

Recent advances in proteomic and immunologic reagents specific for dimethylarginines have contributed to the rapid detection of this post-translational modification in many of the proteins that play a role in mRNP biogenesis. However, identifying which PRMT(s) are responsible for these modifications remains a challenge. This task is not trivial, as the potential for a substrate to be methylated by multiple PRMTs will increase the amount of work that will be required to properly define the specific PRMT(s) for each modification. This scenario is exemplified by the mammalian PABPN1, which is a substrate for multiple PRMTs [110]. Studies in yeast have shown that methylarginines serve as a molecular switch signaling the dissociation of different mRNP components during the progression of mRNP biogenesis. One should also consider the possibility of synergistic regulation between modified histones and RNA processing proteins, given the emerging link between a specific histone modification and its ability to modulate alternative splicing events [101]. Indeed, a number of PRMTs that modify mRNP components have been shown to modify histones [117, 118] 

In conclusion, delineating the molecular mechanisms underlying how arginine methylation influences different steps during mRNP biogenesis would provide significant insight into how this modification shapes the mRNP dynamics. Given the prevalence of arginine methylation in factors that participate at multiple stages of mRNP biogenesis, this modification is likely to contribute to the uniqueness of an “mRNP code” that exists for each transcript in a given cell type, by promoting specific molecular recognition events responsible for the multitude of functional connections. This “code” is dynamic, reflecting the changes that occur as the primary transcript undergoes various processing steps. Future studies employing high-throughput proteomics and genomics technologies are expected to aid in elucidating the impact of this modification on mRNP dynamics.
